# Key ferroptosis-related genes in abdominal aortic aneurysm formation and rupture as determined by combining bioinformatics techniques

**DOI:** 10.3389/fcvm.2022.875434

**Published:** 2022-08-09

**Authors:** Jinrui Ren, Yanze Lv, Lianglin Wu, Siliang Chen, Chuxiang Lei, Dan Yang, Fangda Li, Changzheng Liu, Yuehong Zheng

**Affiliations:** ^1^Department of Vascular Surgery, Peking Union Medical College Hospital, Chinese Academy of Medical Sciences and Peking Union Medical College, Beijing, China; ^2^State Key Laboratory of Complex Severe and Rare Diseases, Peking Union Medical College Hospital, Chinese Academy of Medical Sciences and Peking Union Medical College, Beijing, China; ^3^Chinese Academy of Medical Sciences and Peking Union Medical College, Beijing, China; ^4^Department of Computational Biology and Bioinformatics, Institute of Medicinal Plant Development, Chinese Academy of Medical Sciences and Peking Union Medical College, Beijing, China; ^5^NHC Key Laboratory of Systems Biology of Pathogens, Institute of Pathogen Biology (IPB), Chinese Academy of Medical Sciences (CAMS) and Peking Union Medical College, Beijing, China; ^6^State Key Laboratory of Complex Severe and Rare Disease, Department of Vascular Surgery, Peking Union Medical College Hospital, Chinese Academy of Medical Sciences and Peking Union Medical College, Beijing, China

**Keywords:** AAA (abdominal aortic aneurysm), RAAA, ferroptosis, infiltration of immune cells, GPX4

## Abstract

**Objectives:**

Abdominal aortic aneurysm (AAA) is a cardiovascular disease with high mortality and pathogenesis closely related to various cell death types, e.g., autophagy, apoptosis and pyroptosis. However, the association between AAA and ferroptosis is unknown.

**Methods:**

GSE57691 and GSE98278 dataset were obtained from the Gene Expression Omnibus database, and a ferroptosis-related gene (FRG) set was downloaded from the FerrDb database. These data were normalized, and ferroptosis-related differentially expressed genes (FDEGs, AAA vs. normal samples) were identified using the limma package in R. FRGs expression was analyzed by Gene Set Expression Analysis (GSEA), and FDEGs were analyzed by Gene Ontology (GO) and Kyoto Encyclopedia of Genes (KEGG) pathway enrichment analyses using the clusterProfiler package in R and ClueGO in Cytoscape. Protein–protein interaction networks were assembled using Cytoscape, and crucial FDEGs were identified using CytoHubba. Critical FDEG transcription factors (TFs) were predicted with iRegulon. FDEGs were verified in GSE98278 set, and key FDEGs in AAA (compared with normal samples) and ruptured AAA (RAAA; compared with AAA samples) were identified. Ferroptosis-related immune cell infiltration and correlations with key genes were analyzed by CIBERSORT. Key FEDGs were reverified in Ang II-induced AAA models of ApoE^–/–^ and CD57B/6J mice by immunofluorescence assay.

**Results:**

In AAA and normal samples, 40 FDEGs were identified, and the expression of suppressive FRGs was significantly downregulated with GSEA. For FDEGs, the GO terms were response to oxidative stress and cellular response to external stimulus, and the KEGG pathways were the TNF and NOD-like receptor signaling pathways. IL6, ALB, CAV1, PTGS2, NOX4, PRDX6, GPX4, HSPA5, HSPB1, and NCF2 were the most enriched genes in the crucial gene cluster. CEBPG, NFAT5, SOX10, GTF2IRD1, STAT1, and RELA were potential TFs affecting these crucial genes. Ferroptosis-related immune cells involved in AAA formation were CD8+ T, naive CD4+ T, and regulatory T cells (Tregs); M0 and M2 macrophages; and eosinophils. Tregs were also involved in RAAA. GPX4, SLC2A1, and PEBP1 expression was downregulated in both the RAAA and AAA samples. GPX4 and PEBP1 were more important in AAA because they influenced ferroptosis-related immune cell infiltration, and SLC2A1 was more important in RAAA.

**Conclusions:**

This is the first study to show that ferroptosis is crucial to AAA/RAAA formation. The TNF and NOD-like signaling pathways and ferroptosis-related immune cell infiltration play key roles in AAA/RAAA. GPX4 is a key ferroptosis-related gene in AAA. Ferroptosis and related genes might be promising targets in the treatment of AAA/RAAA.

## Introduction

Abdominal aortic aneurysms (AAAs) occur more frequently in men than women (1), with the prevalence of AAAs in 65-year-old men at 1–2%, and approximately 50% of patients with a ruptured AAA (RAAA) die before receiving medical treatment (2). Histopathological analyses have revealed that AAA is associated with inflammation, smooth muscle cell (SMC) apoptosis and matrix degradation, and AAA risk factors include age, male smoking, and low levels of high-density lipoprotein (HDL) cholesterol (3). The role of inflammation in AAA pathogenesis has been established. Notably, oxidative stress increases in inflammation, playing an important role in the pathophysiology of inflammation (4). In addition, reactive oxygen species (ROS) play key roles in regulating matrix metalloproteinases (MMPs) and inducing SMC apoptosis, and ROS may also contribute to the pathogenesis of hypertension, which is a risk factor for AAA (5). The most widely used classification refers to three types of cell death in animals: apoptosis, necroptosis, and autophagy (6). The importance of cell death in all stages of atherosclerosis is obvious (7). For example, the apoptosis of vascular SMCs (VSMCs) promotes the development of AAAs (8); SMC necrotic apoptosis has been proven to contribute to the AAA pathogenesis (9); autophagy is related to cellular protection against inflammation and oxidative stress, and VSMC autophagy is also involved in AAA formation (10). In our previous studies, macrophage pyroptosis was found to be involved in AAA (11). Therefore, in addition to the inflammatory response, oxidative stress, extracellular matrix degradation and other processes, many cell death modalities play important roles in AAA progression.

Ferroptosis is a regulated form of cell death (12) that depends on iron and causes unrestricted lipid peroxidation and subsequent membrane damage (13). Iron metabolism and lipid peroxidation are increasingly considered to be the central mediators of ferroptosis, which means that ferroptosis plays an important role in regulating oxidative stress and the inflammatory response (14). Accumulating evidence suggests that ferroptosis is involved in diverse cardiovascular diseases, such as atherosclerosis, stroke, ischemia–reperfusion injury and heart failure (15–17). Several epidemiological studies and animal experiments suggest that ferroptosis plays an essential role in the pathophysiology of cardiovascular diseases, such as atherosclerosis (18). Meanwhile, several studies demonstrated the mechanism of ferroptosis in aortic dissection (19, 20). Ferroptosis is a novel and critical pathological mechanism involved in SMC loss and AD development, and Brd4770 is a novel ferroptosis inhibitor with the same protective effect against ferrostatin-1 at optimal concentrations (21).

Most important, ferroptosis plays an important role in cardiovascular disease by participating in inflammatory reactions and oxidative stress, regulating cell death and promoting atherosclerosis.

The relationship between ferroptosis and AAA has not been reported. Recent research suggests that ferroptosis might be involved in AAA formation, which is a potential research target. In this study, we focused on analyzing ferroptosis-related genes (FRGs) that play a role in the AAA formation.

## Materials and methods

### Data acquisition and processing

For this study, gene expression sets were downloaded from the Genes Expression Omnibus database^1^. Specifically, the GSE57691 dataset [including 49 AAA samples 9 arterial occlusive disease (AOD) and 10 donated normal aortic vessel samples] (22) and GSE98278 dataset (including 31 unruptured AAA and 17 RAAA samples). A FRG dataset that included information on 259 genes, was downloaded from the FerrDb database^2^. We used the limma package (23) in R (4.0.5) to correct the sample data.

### Principal component analysis, volcano plots, and heatmaps

After the data were standardized using the limma software package, we identified FRGs and ferroptosis-related differentially expressed genes (FDEGs) by comparing AAA samples with normal samples in the GSE57691 dataset. Genes for which the difference in expression was greater than 1.5-fold (| fold change| ≥ 1.5) and the *P* value (false discovery rate) ≤ 0.05 were considered to be differentially expressed. Volcano plots and heatmaps showing FDEGs were generated using the ggplot2 package in R software. The expression of FDEGs in AAA samples and normal samples was also verified by determining their expression levels in unruptured AAA and RAAA samples in the other dataset (GSE98278).

### Gene ontology and Kyoto encyclopedia of genes and genomes enrichment analyses

Analysis of gene ontology (GO) terms and Kyoto encyclopedia of genes and genomes (KEGG) pathways enriched with FDEGs was performed with the clusterProfiler package (24) in R (4.1.1). Hypergeometric distribution was evaluated to calculate the significance differences in FDEG expression in each signaling pathway. Using this method, we identified the signaling pathways significantly affected by FDEGs (*P* < 0.05).

### Gene set enrichment analysis

Gene set enrichment analysis (GSEA) of the FRGs was performed with the clusterProfiler package R software. The GSEA reference sets were built on the basis of the FRG set. To perform the GSEA, the AAA samples (GSE57691) were divided into two groups (the AAA and normal sample groups). In contrast with conventional GSEA, the grouping of these samples was based on the expression level of each gene in a single-gene analysis.

### Construction and analysis of a protein–protein interaction network, hub genes and transcription factor prediction

The correlations between FDEGs were evaluated by Pearson’s test and mapped using the Corrplot package in R software. Enriched GO and KEGG of FDEGs were mapped with the Cytoscape (3.6.5) ClueGO plug-in. The STRING database [protein–protein interaction (PPI) Networks^3^ ] was used to construct a PPI network with the identified FDEGs. The PPI file was imported into Cytoscape (3.6.5), and the CytoHubba plug-in was used to map the PPIs. The degree, closeness, intermediate degree of each node in the network, and the average value of the nodal degree of each protein were defined to generate the thresholds for inclusion of PPI network nodes, and proteins for which the node degree was greater than the threshold value were selected for inclusion. The transcription factors identified as hub genes were predicted with the iRegulon plug-in (25).

### Infiltration of immune cells in the samples

To investigate the infiltration of immune cells in AAA and normal aortic vessel samples for which FRG expression had been determined, we extracted subsets from the GSE57691 and GSE98278 databases and evaluated and predicted the enrichment of immune cells in the samples. We used the CIBERSORT package (26) in R software. CIBERSORT is a tool used to deconvolute the expression matrix of immune cell subtypes based on the principle of linear support vector regression. The correlations between key FDEGs and immune cells were calculated and mapped using R software. Single genes from among the key genes that correlated with infiltrating immune cells were assessed, and the results are reported.

### Ang II-Induced AAA animal model and aortic tissue collection

ApoE-knockout (ApoE^–/–^) male mice and C57BL/6 male mice were obtained and housed in a pathogen-free barrier facility. AAA was induced in male mice (10–12 weeks old) by the infusion of Ang II (1,000 ng/kg per minute; Sigma–Aldrich, St. Louis, MO, United States) or saline for 28 d through implanted ALZET mini-osmotic pumps (model 2007; DURECT, Cupertino, CA, United States) as described in our previous study (27). The mice were euthanized when 16 weeks old, and further histological and molecular analyses were performed. Abdominal aortic tissues were obtained from AAA and normal mice who had undergone the surgical procedures as previously described. Abdominal aortic tissues were preserved at −80°C or soaked in formalin and embedded in wax blocks. All animal studies were approved by the Institutional Animal Care and Use Committee of Peking Union Medical College Hospital, and experiments conformed to the Guide for the Care and Use of Laboratory Animals [National Institutes of Health (NIH) publication no. 85–23, 1996]. The study protocol was approved by the Ethics Committee of Peking Union Medical College Hospital. All experiments were performed in accordance with the relevant guidelines and regulations.

### Hematoxylin–eosin staining and immunofluorescence

Sections of 5 μm thickness were stained with hematoxylin–eosin (HE) (Gene Pool, GPB1826, China) according to standard pathology-based procedures. For immunofluorescence analysis, cells grown on fibronectin-coated glass coverslips or frozen aortic sections were fixed with 4% paraformaldehyde (PFA) for 15 min, permeabilized with 0.1% Triton X-100, and blocked with 10% goat serum at room temperature for 1 h. The samples were incubated with anti-glutathione peroxidase 4 (GPX4) antibody (1:50, sc-166570, Santa Cruz Biotechnology, United States), anti-solute carrier family 2 member 1 (SLC2A1) antibody (1:200, ab115730, Abcam, United States), and anti-SLC2A1 antibody (1:200, ab76582, Abcam, United States) at 4°C overnight. The next day, the slides were incubated with goat anti-rabbit/rat tetramethylrhodamine (TRITC)/fluorescein isothiocyanate (FITC) secondary antibody for 1 h at room temperature and mounted with DAPI (Beyotime, C1002, Beijing, China). Images were obtained using a confocal microscope (3DHISTECH, Pannoramic MIDI). At least five fields in each coverslip were captured. Image analysis was performed with ImageJ software (NIH, Bethesda, MD, United States).

### Statistical analysis

Data were analyzed using R software (4.1.1). All values are presented as the mean ± SEM of three experimental repeats. Statistical differences between two groups were determined by Student’s *t* test, and for comparisons between three or more groups, ANOVA followed by Bonferroni multiple comparisons was performed. *p* < 0.05 was considered to be statistically significant.

## Results

### The workflow of this study and the FDEGs in the AAA aortic vessel and normal samples

**FIGURE 1:**
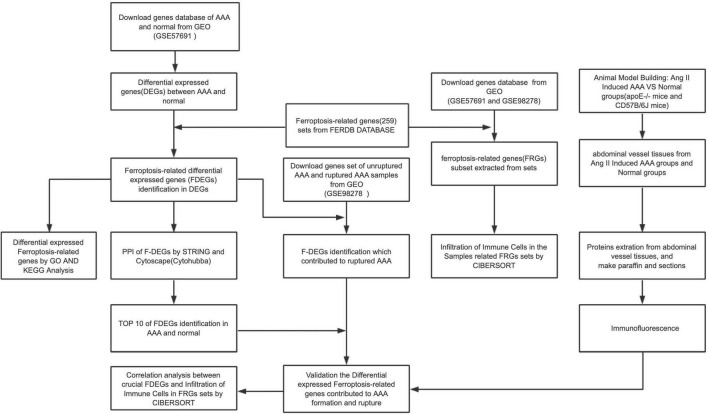
The workflow of this study.

**FIGURE 2:**
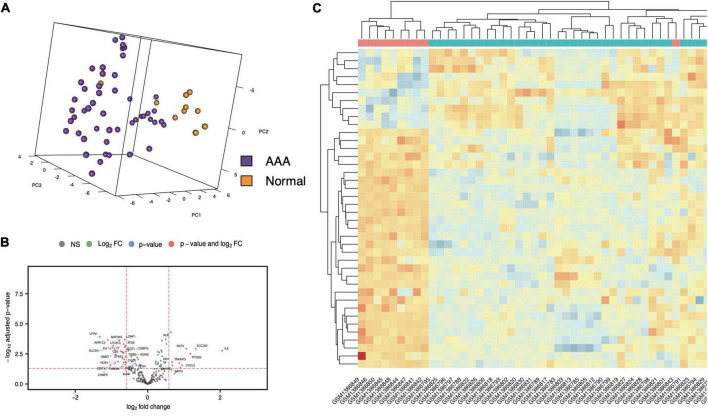
FDEGs in AAAs aortic vessels samples and normal samples of GSE57691. The Principal Component Analysis of AAAs aortic vessels samples and normal samples in GSE57691 set **(A)**. The volcano plots of FDEGs in FRGs subsets of GSE57691 **(B)**. The heatmap of FDEGs **(C)**.

The workflow of the present study is shown in Figure 1. FRG sets were extracted from the GSE57691 dataset (49 AAA and 10 normal samples), and 240 of the 259 FRGs were identified in both GEO sets. Among genes that met the threshold *p* value < 0.05 and | fold-change| > 1.5, in the AAA samples, the expression of 278 FRGs was upregulated, and that of 1,307 FRGs was downregulated (Supplementary Figure 1). We screened 40 FDEGs in the AAA samples, among which the expression of 10 FDEGs was upregulated and that of 30 FDEGs was downregulated compared with their expression in the normal samples (Figures 2A–C and Supplementary Table 1).

### The GSEA of each gene

A GSEA was performed for each crucial gene (Supplementary Figure 2). Gene sets associated with suppressive FRGs were significantly downregulated in the AAA group, which suggested that ferroptosis plays important roles in AAA progression.

### GO and KEGG analyses of the FDEGs in the AAA aortic vessel and normal samples

**FIGURE 3:**
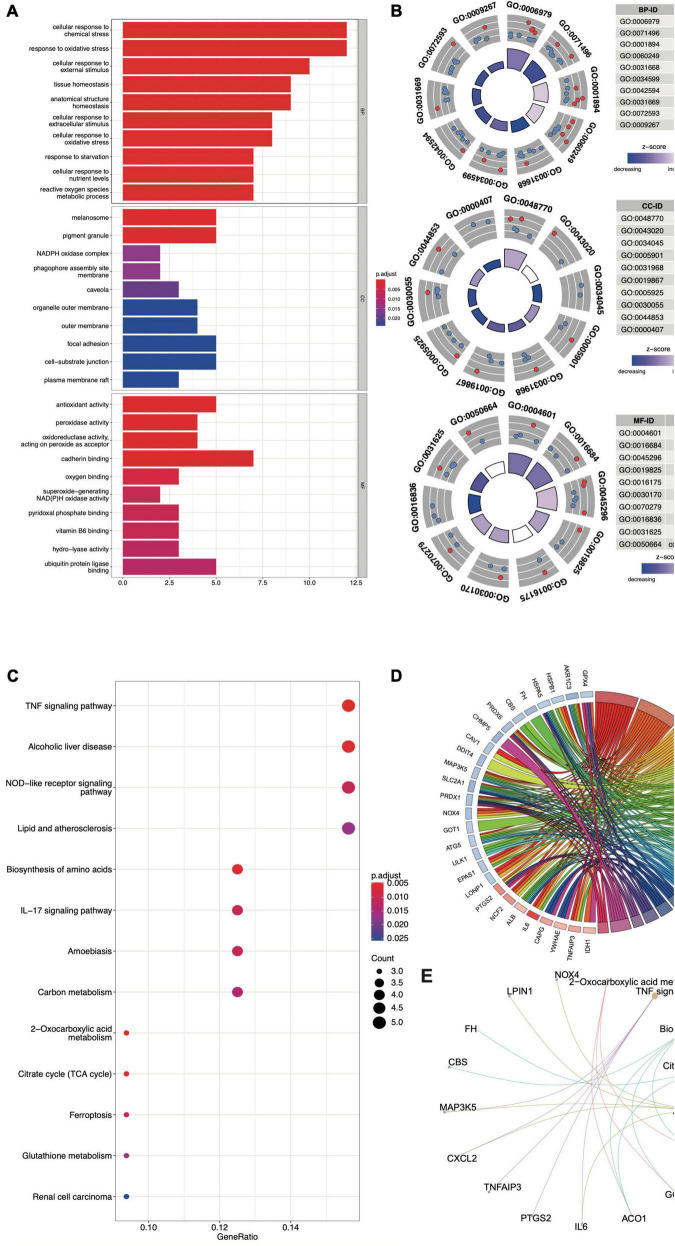
The GO and KEGG analysis of FDEGs in AAAs aortic vessels samples and normal samples of GSE57691. The GO-BP, CC, and MF analysis of FDEGs **(A,B)**. the KEGG analysis of FDEGs **(C)**. The top 5 enriched GO **(D)** and KEGG **(E)** pathway of FDEGs.

The biological functions of the FDEGs in AAA and normal samples were identified through GO and KEGG pathway analyses (Figure 3 and Supplementary Tables 2, 3). The crucial FDEG-enriched GO biological processes (BPs) were response to oxidative stress and cellular response to external stimulus, and the GO cell components (CCs) were pigment granules and NADPH oxidase complexes, while the GO molecular functions (MFs) of the enriched FDEGs were peroxidase activity and oxidoreductase activity (Figures 3A,B). The crucial KEGG pathways were the TNF signaling pathway, alcoholic liver disease, NOD-like receptor signaling pathway, lipid and atherosclerosis, and biosynthesis of amino acids (Figure 3C). GO terms and KEGG pathways enriched with the highest significantly adjusted *p* values are shown in Table 1 and Figures 3D,E. These results suggested that responses to ferroptosis-related inflammation and oxidative stress might play roles in AAA formation.

**TABLE 1:** Top 5 GO and KEGG pathways enriched FDEGs counts FDEGs in AAA and normal aortic samples.

ID	Description	Counts
**TOP 5-GO**		
GO:0062197	Cellular response to chemical stress	14
GO:0006979	Response to oxidative stress	14
GO:0071496	Cellular response to external stimulus	11
GO:0001894	Tissue homeostasis	9
GO:0060249	Anatomical structure homeostasis	9
**TOP 5-KEGG**		
hsa04668	TNF signaling pathway	5
hsa04936	Alcoholic liver disease	5
hsa04621	NOD-like receptor signaling pathway	5
hsa05417	Lipid and atherosclerosis	5
hsa01230	Biosynthesis of amino acids	4

### Construction and analysis of the protein–protein interaction network, hub genes and transcription factor predictions related to FDEGs involved in AAA formation

**FIGURE 4:**
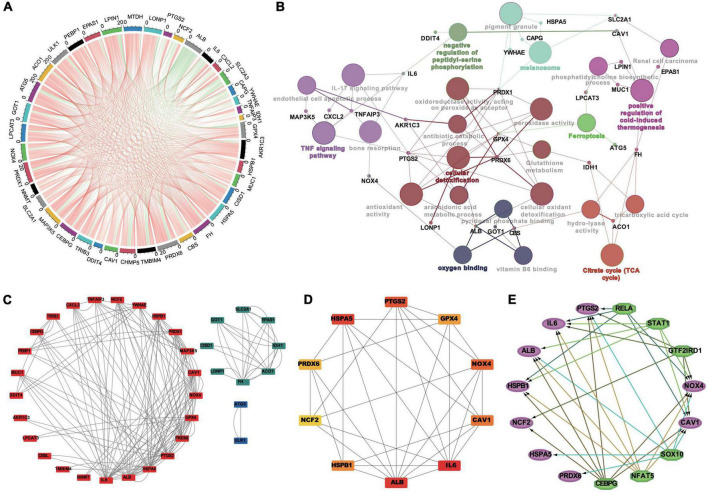
The correlation analysis, the PPI networks and TFs prediction of FDEGs in AAAs aortic vessels samples and normal samples of GSE57691. The Pearson’s correlation analysis in FDEGs **(A)**. The relationships of pathways and FDEGs using the Clue GO analysis **(B)**. The function clusters of FDEGs using the MCL of Cytoscape **(C)**. The top 10 hub genes of FDEGs using the Cytohubba of Cytoscape **(D)**. The TFs of the hub FDEGs predicted by the iRegulon of Cytoscape **(E)**.

The correlation of FDEGs was calculated on the basis of their expression in AAA aortic vessel and normal samples (Figure 4A). An FDEG coexpression network including GO terms and KEGG pathways (cellular detoxification, positive regulation of cold-induced thermogenesis, the TNF signaling pathway, etc.) was generated with the Cytoscape ClueGO plug-in (Figure 4B). After removing isolated nodes and node pairs, the network included 190 edges and 35 nodes (Figure 4C). The maximal clique centrality (MCC) method was used (with the Cytoscape plug-in CytoHubba) to generate a subnetwork with 10 crucial genes (Figure 4D and Table 2). IL6, ALB, CAV1, PTGS2, NOX4, PRDX6, GPX4, HSPA5, HSPB1, and NCF2 showed the highest degrees of centrality in the crucial gene cluster. CEBPG, NFAT5, SOX10, GTF2IRD1, STAT1, and RELA were identified TFs that may affect these crucial genes (Figure 4E).

**TABLE 2:** The crucial FDEGs in AAA and normal aortic samples.

Gene	Protein names	AAA
IL6	Interleukin-6	+
ALB	Albumin	+
CAV1	Caveolin-1	–
PTGS2	Prostaglandin G/H synthase 2	+
NOX4	NADPH oxidase 4	–
PRDX6	Peroxiredoxin-6	–
GPX4	Phospholipid hydroperoxide glutathione peroxidase	–
HSPA5	Endoplasmic reticulum chaperone BiP	–
HSPB1	Heat shock protein beta-1	–
NCF2	Neutrophil cytosol factor 2	+

+, Up-regulated expression; –, Down-regulated expression.

### Expression verification of the FDEGs in the RAAA and unruptured AAA samples

We screened 40 FDEGs that showed significantly different expression levels in the AAA and normal aortic wall samples. Thirteen FDEGs in the GSE98278 dataset (17 RAAA and 31 AAA samples) were differentially expressed; the expression of 7 FDEGs was upregulated, and that of 6 FDEGs was downregulated (Table 3 and Supplementary Figure 3). Three of these 13 genes exhibited similar expression patterns; the expression of GPX4, phosphatidylethanolamine-binding protein 1 (PEBP1), and SLC2A1 was downregulated in the RAAA samples compared with that in the unruptured AAA samples, and their expression was downregulated in the AAA samples compared with that in the normal aortic wall samples.

**TABLE 3:** The FDEGs in GSE57691 and GSE98278.

Gene name	Protein name	RAAA vs AAA	AAA vs normal
LPCAT3	Lysophospholipid acyltransferase 5	+	–
ACO1	Cytoplasmic aconitate hydratase	+	–
ULK1	Serine/threonine-protein kinase ULK1	+	–
PEBP1	Phosphatidylethanolamine-binding protein 1	–	–
LONP1	Lon protease homolog, mitochondrial	+	–
CAPG	Macrophage-capping protein	–	+
GPX4	Phospholipid hydroperoxide glutathione peroxidase	–	–
HSPA5	Endoplasmic reticulum chaperone BiP	+	–
CBS	Cystathionine beta-synthase	+	–
PRDX6	Peroxiredoxin-6	+	–
TMBIM4	Protein lifeguard 4	–	–
DDIT4	DNA damage-inducible transcript 4 protein	+	–
SLC2A1	Solute carrier family 2	–	–

### Ferroptosis-related immune cell infiltration in the samples

**FIGURE 5:**
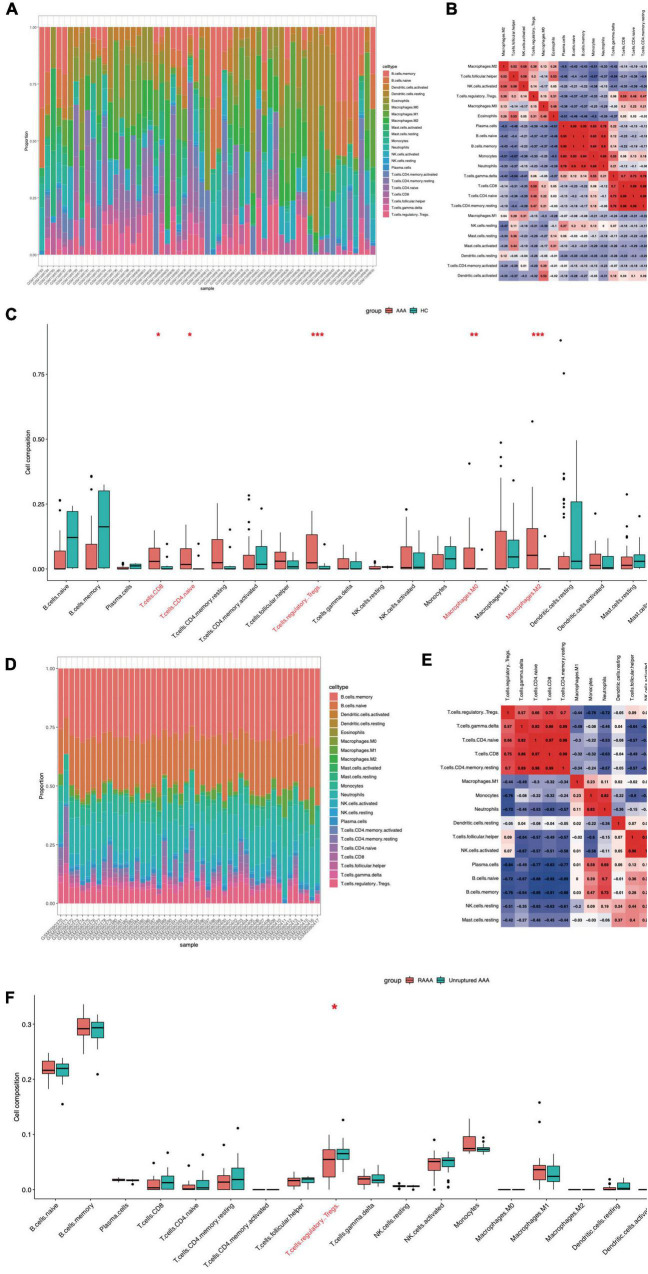
Ferroptosis-related infiltration of immune cells in the FEGs subsets of GSE57691 and GSE98278. The infiltration of immune cells in FEGs subsets of GSE57691 **(A)**. The correlation of immune cells in FEGs subsets of GSE57691 **(B)**. The difference infiltration of immune cells in FEGs subsets of GSE57691 **(C)**. The infiltration of immune cells in FEGs subsets of GSE98278 **(D)**. The correlation of immune cells in FEGs subsets of GSE98278 **(E)**. The difference infiltration of immune cells in FEGs subsets of GSE98278 **(F)**.

We used the CIBERSORT package (R software) to analyze immune cell infiltration in the samples, and 49 AAA samples and 10 normal aortic samples extracted from the GSE57691 dataset that met the standard for FRG expression were selected for this analysis (*P* < 0.05). We found a significant difference in the abundance of 22 types of immune cells between the 10 normal aortic samples and 49 AAA samples (Figure 5A). Specifically, we found significant differences in the infiltration ratio of CD8+ T cells, naive CD4+ T cells, regulatory T cells (Tregs), M0 macrophages, M2 macrophages and eosinophils between the normal aortic and AAA samples (Figure 5C). Among all the differentially abundant immune cells in the AAA and normal samples, naive CD4+ T cells and CD8+ T cells were the most positively correlated (Pearson’s correlation = 0.99), and the second strongest positive correlation was found for CD8+ T cells and Tregs (Pearson’s correlation = 0.59) (Figure 5B). In addition to these infiltrated immune cell samples, we selected 31 unruptured AAA samples and 17 RAAA samples extracted from GSE98278 dataset because they met the FRG expression standard, and a Pearson’s test was performed with these samples to determine correlations (Figure 5D). The infiltration ratio of Tregs was significantly different in the RAAA samples (Figure 5F). Specifically, plasma cells were the most negatively correlated with Tregs (Pearson’s correlation = −0.84), while CD8+ T cells and Tregs were most positively correlated in the RAAA and unruptured AAA samples (Pearson’s correlation = 0.75) (Figure 5E).

### Correlations of infiltrating immune cells with GPX4, SLC2A1, and PEBP1 expression in the normal, AAA and RAAA samples

**FIGURE 6:**
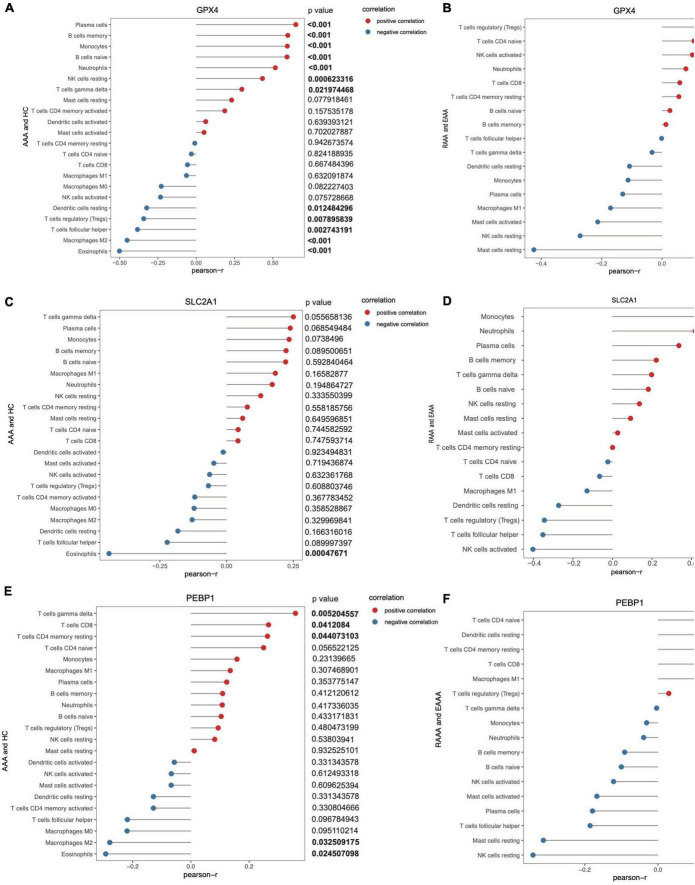
The correlation of key FDEGs and infiltration of immune cells in the FRGs subsets of GSE57691 and GSE98278. The correlation of GPX4 and infiltration of immune cells in AAA and normal samples of GSE57691 **(A)**. The correlation of GPX4 and infiltration of immune cells in RAAA and AAA samples of GSE98278 **(B)**. The correlation of SLC2A1 and infiltration of immune cells in AAA and normal samples of GSE57691 **(C)**. The correlation of SLC2A1 and infiltration of immune cells in RAAA and AAA samples of GSE98278 **(D)**. The correlation of PEBP1 and infiltration of immune cells in AAA and normal samples of GSE57691 **(E)**. The correlation of PEBP1 and infiltration of immune cells in RAAA and AAA samples of GSE98278 **(F)**.

Correlations between GPX4 expression and immune cells in the AAA and normal aortic vessel samples (the GSE57691 dataset) and in the RAAA and unruptured AAA samples (the GSE98278 dataset) were determined. GPX4 expression was positively correlated with plasma cells, memory B cells, monocytes, naïve B cells, neutrophils, resting natural killer (NK) cells, and gamma delta T cells, while resting dendritic cells, Tregs, follicular helper T cells, M2 macrophages, and eosinophils were negatively correlated with GPX4 expression in the AAA and normal aortic vessel samples (*p* value < 0.05, Figure 6A). GPX4 was negatively correlated with resting mast cells in the RAAA and unruptured AAA samples (*p* value < 0.05, Figure 6B). The correlations between GPX4 expression and infiltrating immune cells in single-gene groups are shown in Supplementary Figures 4A–D. SLC2A1 expression was only negatively correlated with eosinophils in the AAA and normal aortic vessel samples. However, SLC2A1 expression was positively correlated with monocytes, neutrophils, and plasma cells in the RAAA and unruptured AAA samples, and it was negatively correlated with Tregs, follicular helper T cells, and activated NK cells in the AAA samples (Figures 6C,D). The correlations between SLC2A1 expression and infiltrating immune cells in the single-gene groups are shown in Supplementary Figures 4E–H. PEBP1 expression was positively correlated with gamma delta T cells, CD8+ T cells, and resting memory CD4+ T cells in the AAA and normal aortic vessel samples (Figure 6E) and negatively correlated with M2 macrophages and eosinophils. PEBP1 expression was negatively correlated with neutrophils, activated mast cells, and resting mast cells in the RAAA and unruptured AAA samples (Figure 6F).

### The expression of key FDEGs was verified in Ang II-induced AAA model mice

**FIGURE 7:**
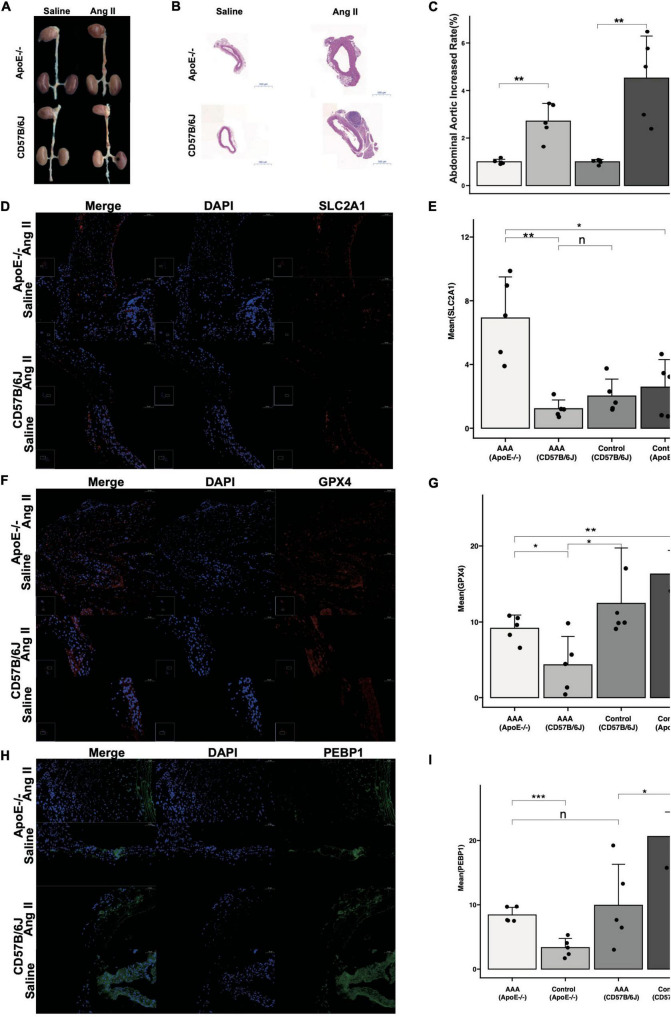
The reverified of key FDEGs with Ang II induced-AAA model in ApoE^–/–^ and CD57B/6J mice. The drams of abdominal aorta in groups **(A)**. The HE staining of abdominal aorta in each group **(B)**. The abdominal aorta diameters increased rate of AAA compared with control samples [**(C)**, *n* = 5]. The IF of SLC2A1 on abdominal aorta in groups **(D)** and mean area of SLC2A1 in groups **(E)**. The IF of GPX4 on abdominal aorta in groups **(F)** and mean area of GPX4 in groups **(G)**. The IF of PEBP1 on abdominal aorta in groups **(H)** and mean area of PEBP1 in groups **(I)** (n: *p* > 0.05; *p < 0.05; **p < 0.01; ***p < 0.001).

After 28 d of Ang II treatment in mice, the mouse aortas showed significant expansion compared with those in mice treated with saline (Figures 7A,C). HE staining revealed that Ang II alleviated luminal region expansion, media thinning, and adventitia thickening (Figure 7B). SLC2A1 expression was upregulated to a greater degree in the AAA tissues compared than in normal aortic walls of ApoE^–/–^ models, and it was downregulated in the AAA tissues compared than in normal aortic walls of CD57B/6J models (Figures 7D,E). GPX4 expression was downregulated to a greater degree in the AAA tissues than in normal aortic walls in ApoE^–/–^ and CD57B/6J models (Figures 7F,G). PEBP1 expression was upregulated to a greater degree in the AAA tissues compared than in normal aortic walls of ApoE^–/–^ models, and it was downregulated in the AAA tissues compared than in normal aortic walls of CD57B/6J models (Figures 7H,I).

## Discussion

In the present study, key genes involved in ferroptosis were identified, and the mechanisms of ferroptosis in AAA formation and AAA rupture were explored. Correlations between FRGs and immune cells in AAA formation and AAA rupture were also determined, for the first time, in this study. Moreover, we described 40 FDEGs identified from among the FDEGs in both the GSE57691 and FerrDb datasets. These FDEGs included 30 with downregulated expression and 10 with upregulated expression in the AAA samples compared with that in normal aortic vessel samples. Then, pathway enrichment analysis with these FDEGs was performed. The 5 most enriched GO terms were cellular response to chemical stress, response to oxidative stress, cellular response to external stimulus, tissue homeostasis, and anatomical structure homeostasis. The 5 most enriched KEGG pathways were the TNF signaling pathway, alcoholic liver disease, NOD-like receptor signaling pathway, lipid and atherosclerosis, and biosynthesis of amino acids. IL6, ALB, CAV1, PTGS2, NOX4, PRDX6, GPX4, HSPA5, HSPB1, and NCF2 were the crucial FDEGs involved in AAA formation. The FDEGs that contributed to AAA formation were also verified in the RAAA and AAA samples extracted from the GSE98278 database. However, GPX4, SLC2A1, and PEBP1 expression was downregulated in the RAAA samples compared with that in the unruptured AAA samples, and their expression was downregulated in the AAA samples compared with that in the normal aortic wall samples. In addition, among the immune cells that infiltrated the AAA samples (compared with normal samples) and RAAA samples (compared with AAA samples) with FDEG expression that met the criteria for selection, the numbers of CD8+ T cells, naive CD4+ T cells, Tregs, M0 macrophages, M2 macrophages and eosinophils were higher in the AAA samples than in the normal samples, and fewer Tregs were found in the RAAA sample than in the AAA sample. Single-gene correlation analysis was performed with the FDEG expression subsets extracted from the GSE57691 or GSE98278 datasets. GPX4 expression was highly correlated with immune cell abundances in the AAA and normal samples, while SLC2A1 expression was more closely related to RAAA. This study may provide a useful reference for studying the pathological mechanisms of AAA and RAAA from the perspective of bioinformatics.

Ferroptosis is characterized by the intracellular abundance of lipid ROS, which are related and result in the lipid oxidation, leading to cell membrane injury and cell death (12, 13, 28). Ferroptosis is associated with various pathological conditions, including acute tissue injury, infection, cancer, and neurodegeneration (29). Ferroptosis also plays an important role in cardiovascular diseases, such as atherosclerosis, coronary heart disease, and cardiac hypertrophy (17, 30, 31). Moreover, ferroptosis is closely related to the inflammatory response and oxidative stress in vascular endothelial cells, SMCs, and macrophages (17, 32, 33). In this study, we found that FDEGs were mostly enriched in the response to oxidative stress, which includes IL6, PTGS2, PRDX6, GPX4, and HSPB1. As in previous studies, ferroptosis was found to be closely related to ROS, and ROS are crucial to AAA (14, 34). Ferroptosis-inducing factors can directly or indirectly affect glutathione peroxidase through different pathways, resulting in decreased intracellular antioxidant capacity and lipid ROS accumulation, which ultimately leads to oxidative stress-related cell death (29). ROS and oxidative stress promote the degradation of the extracellular matrix (ECM) and apoptosis of VSMCs and promote the formation and progression of AAA (5, 35). However, few studies have reported correlations between lipid peroxidation and AAA, which might become a new research direction. After oxidative stress, some signaling pathways were found to be enriched with FDEGs, such as the TNF pathway and NOD-like receptor signaling pathway. Cigarette smoke extract has been shown to upregulate TNF-α, MMP-2, and MMP-9 expression in SMCs, and this effect was inhibited by Fer-1 (a ferroptosis inhibitor) (33). TNF-α signaling-induced oxidative stress and apoptosis have been implicated in AAA pathogenesis (36, 37). The core role of the Nod-like receptor (NLR) protein family in the innate immune response involves the signal transduction ATP enzyme, which affects atherosclerosis and AAA formation by modulating inflammation that can cause autoimmunity (31, 38, 39). NLRs also play roles in ferroptosis by regulating oxidative stress (40). Therefore, FRGs may be involved in AAA formation by regulating the TNF and NLR signaling pathways and regulating the oxidative stress and inflammatory reaction in the cells of the aortic wall, such as endothelial cells, SMCs, and macrophages. Regulating FRG expression might reduce oxidative stress and the inflammatory response associated with AAA, thereby reducing AAA formation.

Immune cells in the blood infiltrate tissues, and these immune cells can be isolated from the tissues they infiltrate. Notably, immune cell infiltration is closely related to clinical outcomes of carcinomas (41, 42). In recent decades, studies have shown that many inflammatory cells, including T cells, macrophages, dendritic cells, neutrophils, B cells and mast cells, infiltrate the aortic wall, suggesting that they may play key roles in AAA formation (43). In a previous study, a higher proportion of macrophages, CD8+ T cells, and resting mast cells was found in AAA tissues than in normal tissues (44). In our study, we found ferroptosis-related differences in the infiltration of immune cells, and Tregs, CD8+ T cells, M0 macrophages, M2 macrophages, eosinophils and naive CD4+ T cells were found in higher proportions in the AAA tissue than in the normal tissue samples. T cells constitute the main leukocyte subset in AAA, and they aggregate in the highest level in perivascular adipose tissue. CD4+ and CD8+ T cell populations have been shown to be highly activated, and CD4+ T cells have been shown to be in the highest activation state in AAA wall (45). In the present study, Tregs were more abundant in unruptured AAA samples than in RAAA samples. Tregs may be a potential therapeutic target for inhibiting AAA progression (46). Several studies have found that Tregs can inhibit AAA formation (47, 48). Tregs cells could suppress the COX-2 expression (49). Simvastatin treatment prevented the development of Ang II induced AAA in ApoE^(–/–)^ mice, which may be partially due to the induction of Treg accumulation (50). In this study, we examined the correlation and differential enrichment of immune cell infiltration with AAA, rAAA, by extracting data subsets of ferroptosis related genes, which was quietly differential with previous study and indicated the relationships in ferroptosis related genes, immune cells infiltration and AAA formation. We found Tregs was higher in AAA samples compared with health samples, while it was higher in AAA compared with rAAA in ferroptosis related genes subsets. And GPX4 was negatively related with Tregs in AAA and healthy samples, which was needed more researches.

Glutathione peroxidase 4 (GPX4), an essential antioxidant peroxidase that directly reduces phospholipid hydroperoxides even when they are incorporated into membranes and lipoproteins, reduces fatty acid hydroperoxides, cholesterol hydroperoxides, and thymine hydroperoxides. It also plays a key role in protecting cells from oxidative damage by preventing membrane lipid peroxidation. GPX4 may be required to prevent cells from undergoing ferroptosis, a non-apoptotic cell death caused by iron-dependent accumulation of lipid ROS (51), and may protect cells from the toxicity induced by ingested lipid hydroperoxides. GPX4 plays a role in primary T cell responses to viral and parasitic infections by protecting T cells from ferroptosis and supporting T cell expansion. It also plays a role in arachidonic acid metabolism in platelets (52). Iron-catalyzed disorder of intracellular lipid peroxide metabolism leads to GPX4 inactivation, destruction of the redox balance, and ferroptosis; these findings show that GPX4 plays an important role in the pathophysiology of cardiovascular diseases such as atherosclerosis, cardiac hypertrophy, cardiomyopathy, and AAA (18, 53). GPX4 is the essential antioxidant enzyme that protects against lipid peroxidation and is a regulator of iron concentration in vascular endothelial cells (32, 54). In our study, we found that GPX4 was involved in AAA formation by regulating immune cell infiltration, and GPX4 expression was most positively correlated with plasma cells and negatively correlated with eosinophils. GPX4 expression was also negatively correlated with resting mast cells in RAAA.

Solute carrier family 2 member 1 (SLC2A1), also known as glucose transporter 1 (GLUT1), enables glucose (a hydrophilic molecule) to move through the cell membrane, and it can promote glycolysis and endothelial cell proliferation and inhibit atherosclerotic lesion formation in hyperlipidemic mice (55). SLC2A1-mediated glucose uptake promotes glycolysis, thereby promoting pyruvate oxidation and the three-ring acid cycle, stimulating fatty acid synthesis, and ultimately promoting lipid peroxidation-dependent ferroptosis (56). The relationship between SLC2A1 expression and AAA is not clear. In our study, we found that SLC2A1 expression was closely correlated with the infiltration of immune cells, especially in RAAA tissues. In FRG subsets extracted from the GSE57691 dataset, SLC2A1 expression was most negatively related with eosinophils. However, SLC2A1 expression was most positively correlated with monocytes and most negatively correlated with activated NK cells in FRG subsets of GSE98278.

PEBP1 (phosphatidylethanolamine-binding protein 1), also called Raf kinase inhibitor protein (RKIP), inhibits RAF1 kinase activity by inhibiting RAF1 activation, causing RAF1/MEK complex dissociation and competitively inhibiting MEK phosphorylation (57). RKIP negatively regulates NLRP1 activation and NLRP3 and NLRC4-induced inflammatory body formation and plays an important role in the pathogenesis of type 2 diabetes (58). Overexpression of CUG triplet repeat-binding protein 1 (CELF1) negatively regulates the protein expression of PEBP1, and knocking down PEBP1 expression partially enhances ROS production and the apoptosis rate of neonatal cardiomyocytes (59). PEBP1 is involved in regulating endothelial cell autophagy and affects atherosclerosis (60). However, the impact of PEBP1 on AAA has not been clearly reported. We found that PEBP1 expression was downregulated to a greater extent in AAA tissues than in normal samples and downregulated in RAAA to a greater extent than in AAA samples. These results indicate that PEBP1 is a crucial factor that negatively regulates AAA formation and suppresses AAA rupture.

Most important, among the types of cell death, ferroptosis is more extensively involved in AAA formation and AAA rupture. Inflammation and the oxidative response are crucial biological processes in AAA formation and AAA rupture, and these processes are highly correlated with ferroptosis. FDEGs were enriched in TNF- and NOD-like signaling pathways in AAA formation. GPX4, SLC2A1 and PEBP1 expression was downregulated in both the AAA and RAAA samples. Differential expression of FRGs resulted in differences in immune cell infiltration in AAA formation and AAA rupture. According to the correlation analysis of single-gene and immune cell infiltration in the FRG subsets, GPX4 and PEBP1 was a more important contributor to AAA formation, and SLC2A1 was a more important contributor to RAAA. According to the reverified with Ang II- induced AAA models, the key FDEG, GPX4, was downregulated in AAA compared to control groups of ApoE^–/–^ and CD57B/6J mice. However, the SLC2A1 and PEBP1 expression were upregulated in AAA models compared with control samples in ApoE^–/–^ mice, while they were downregulated expression in AAA models compared with control samples of CD57B/6J mice. This suggests that the CD57B/6J mouse model, might be better choices for studying the mechanism of ferroptosis involved in AAA formation. This may be related to the knockout of ApoE gene, and further research is needed to prove its specific mechanism.

Some limitations of this study need to be noted. First, the key FDEGs we identified were validated with animal models, but the specific pathways were not confirmed with *in vitro* studies or other functional studies, which will be important experiments in future research. Second, we found that the expression of these key genes was significantly different in RAAA, but because of a lack of animal models and clinical samples, we could not verify these FDEGs. Further study may lead to explanations of the specific mechanisms of these genes in AAA rupture. However, our results can be enlightening, to some extent, in subsequent mechanistic studies.

## Conclusion

Our study was the first to show that ferroptosis plays a crucial role in AAA formation and rupture and that these processes are related to TNF and the NOD-like signaling pathway regulation of inflammatory and oxidative responses. Notably, differential expression of FRGs leads to differences in immune cell infiltration in AAA formation and rupture. The key FRGs, GPX4 may be novel therapeutic target in AAA treatment.

## Data availability statement

The datasets presented in this study can be found in online repositories. The names of the repository/repositories and accession number(s) can be found below: https://www.ncbi.nlm.nih.gov/geo/, GSE57691; https://www.ncbi.nlm.nih.gov/geo/, GSE98278.

## Ethics statement

The animal study was reviewed and approved by the Institutional Animal Care and Use Committee of Peking Union Medical College Hospital.

## Author contributions

JR, YL, and YZ conceived the focus of the study, wrote, and edited the manuscript. JR, YL, LW, and SC contributed expertise with preparation of the manuscript and the figures. CXL, DY, FL, and CZL provided the critical revision with insightful and constructive comments to improve the manuscript. JR and YL contributed equally to this study and manuscript. All authors read and approved the manuscript.

## Footnotes

1
https://www.ncbi.nlm.nih.gov/geo/

2
http://www.zhounan.org/ferrdb/

3
http://string-db.org

## References

[1] Norman PE Powell JT. Abdominal aortic aneurysm: The prognosis in women is worse than in men. *Circulation.* (2007) 115:2865–9. 10.1161/CIRCULATIONAHA.106.671859 17548742

[2] Sakalihasan N Michel JB Katsargyris A Kuivaniemi H Defraigne JO Nchimi A Abdominal aortic aneurysms. *Nat Rev Dis Primers.* (2018) 4:34. 10.1038/s41572-018-0030-7 30337540

[3] Nordon IM Hinchliffe RJ Loftus IM Thompson MM. Pathophysiology and epidemiology of abdominal aortic aneurysms. *Nat Rev Cardiol* (2011) 8:92–102. 10.1038/nrcardio.2010.180 21079638

[4] Chen Q Wang Q Zhu J Xiao Q Zhang L. Reactive oxygen species: Key regulators in vascular health and diseases. *Br J Pharmacol.* (2018) 175:1279–92. 10.1111/bph.13828 28430357 PMC5867026

[5] Mccormick ML Gavrila D Weintraub NL. Role of oxidative stress in the pathogenesis of abdominal aortic aneurysms. *Arterioscler Thromb Vasc Biol* (2007) 27:461–9. 10.1161/01.ATV.0000257552.94483.14 17218601

[6] Richard S Hotchkiss AS Mcdunn JE Swanson PE. Cell death. *N Engl J Med.* (2009) 361:1570–83. 10.1056/NEJMra0901217 19828534 PMC3760419

[7] Rayner KJ. Cell death in the vessel wall: The good, the bad, the ugly. *Arterioscler Thromb Vasc Biol.* (2017) 37:e75–81. 10.1161/ATVBAHA.117.309229 28637702 PMC5584709

[8] Chai H Tao Z Qi Y Qi H Chen W Xu Y IKK epsilon deficiency attenuates angiotensin II-induced abdominal aortic aneurysm formation in mice by inhibiting inflammation, oxidative stress, and apoptosis. *Oxid Med Cell Longev.* (2020) 2020:3602824. 10.1155/2020/3602824 32064021 PMC6998751

[9] Zhou T Deroo E Yang H Stranz A Wang Q Ginnan R MLKL and CaMKII are involved in RIPK3-mediated smooth muscle cell necroptosis. *Cells.* (2021) 10:2397. 10.3390/cells10092397 34572045 PMC8471540

[10] Qin Y Zheng B Yang GS Yang HJ Zhou J Yang Z Salvia miltiorrhiza-derived sal-miR-58 induces autophagy and attenuates inflammation in vascular smooth muscle cells. *Mol Ther Nucleic Acids.* (2020) 21:492–511. 10.1016/j.omtn.2020.06.015 32679544 PMC7360890

[11] Zhang X Li F Wang W Ji L Sun B Xiao X Macrophage pyroptosis is mediated by immunoproteasome subunit β5i (LMP7) in abdominal aortic aneurysm. *Biochem Biophys Res Commun.* (2020) 533:1012–20. 10.1016/j.bbrc.2020.09.082 33019975

[12] Hadian K Stockwell BR. SnapShot: Ferroptosis. *Cell.* (2020) 181:1188–1188e1. 10.1016/j.cell.2020.04.039 32470402 PMC8157339

[13] Tang D Kroemer G. Ferroptosis. *Curr Biol.* (2020) 30:R1292–7. 10.1016/j.cub.2020.09.068 33142092

[14] Yu Y Yan Y Niu F Wang Y Chen X Su G Ferroptosis: A cell death connecting oxidative stress, inflammation and cardiovascular diseases. *Cell Death Discov.* (2021) 7:193. 10.1038/s41420-021-00579-w 34312370 PMC8313570

[15] Kobayashi M Suhara T Baba Y Kawasaki NK Higa JK Matsui T. Pathological roles of iron in cardiovascular disease. *Curr Drug Targets.* (2018) 19:1068–76. 10.2174/1389450119666180605112235 29874997 PMC6469984

[16] Chen X Li X Xu X Li L Liang N Zhang L Ferroptosis and cardiovascular disease: Role of free radical-induced lipid peroxidation. *Free Radic Res.* (2021) 55:405–15. 10.1080/10715762.2021.1876856 33455488

[17] Ouyang S You J Zhi C Li P Lin X Tan X Ferroptosis: The potential value target in atherosclerosis. *Cell Death Dis.* (2021) 12:782. 10.1038/s41419-021-04054-3 34376636 PMC8355346

[18] Zhang Y Xin L Xiang M Shang C Wang Y Wang Y The molecular mechanisms of ferroptosis and its role in cardiovascular disease. *Biomed Pharmacother.* (2022) 145:112423. 10.1016/j.biopha.2021.112423 34800783

[19] Zou HX Qiu BQ Lai SQ Huang H Zhou XL Gong CW Role of ferroptosis-related genes in Stanford type a aortic dissection and identification of key genes: New insights from bioinformatic analysis. *Bioengineered.* (2021) 12:9976–90. 10.1080/21655979.2021.1988840 34652258 PMC8809966

[20] Chen Y He Y Wei X Jiang DS. Targeting regulated cell death in aortic aneurysm and dissection therapy. *Pharmacol Res.* (2022) 176:106048. 10.1016/j.phrs.2021.106048 34968685

[21] Chen Y Yi X Huo B He Y Guo X Zhang Z BRD4770 functions as a novel ferroptosis inhibitor to protect against aortic dissection. *Pharmacol Res.* (2022) 177:106122. 10.1016/j.phrs.2022.106122 35149187

[22] Biros E Gabel G Moran CS Schreurs C Lindeman JH Walker PJ Differential gene expression in human abdominal aortic aneurysm and aortic occlusive disease. *Oncotarget.* (2015) 6:12984–96. 10.18632/oncotarget.3848 25944698 PMC4536993

[23] Ritchie ME Phipson B Wu D Hu Y Law CW Shi W limma powers differential expression analyses for RNA-sequencing and microarray studies. *Nucleic Acids Res.* (2015) 43:e47. 10.1093/nar/gkv007 25605792 PMC4402510

[24] Yu G Wang LG Han Y He QY. clusterProfiler: An R package for comparing biological themes among gene clusters. *OMICS.* (2012) 16:284–7. 10.1089/omi.2011.0118 22455463 PMC3339379

[25] Chen HZ Wang F Gao P Pei JF Liu Y Xu TT Age-associated sirtuin 1 reduction in vascular smooth muscle links vascular senescence and inflammation to abdominal aortic aneurysm. *Circ Res.* (2016) 119:1076–88. 10.1161/CIRCRESAHA.116.308895 27650558 PMC6546422

[26] Newman AM Liu CL Green MR Gentles AJ Feng W Xu Y Robust enumeration of cell subsets from tissue expression profiles. *Nat Methods.* (2015) 12:453–7. 10.1038/nmeth.3337 25822800 PMC4739640

[27] Li FD Nie H Tian C Wang HX Sun BH Ren HL Ablation and inhibition of the immunoproteasome catalytic subunit LMP7 attenuate experimental abdominal aortic aneurysm formation in mice. *J Immunol.* (2019) 202:1176–85. 10.4049/jimmunol.1800197 30642978

[28] Zhou B Liu J Kang R Klionsky DJ Kroemer G Tang D. Ferroptosis is a type of autophagy-dependent cell death. *Semin Cancer Biol.* (2020) 66:89–100. 10.1016/j.semcancer.2019.03.002 30880243

[29] Li J Cao F Yin HL Huang ZJ Lin ZT Mao N Ferroptosis: Past, present and future. *Cell Death Dis.* (2020) 11:88. 10.1038/s41419-020-2298-2 32015325 PMC6997353

[30] Zhang X Zheng C Gao Z Chen H Li K Wang L SLC7A11/xCT Prevents Cardiac Hypertrophy by Inhibiting Ferroptosis. *Cardiovasc Drugs Ther.* (2021) 36:437–47. 10.1007/s10557-021-07220-z 34259984

[31] Zhou Y Zhou H Hua L Hou C Jia Q Chen J Verification of ferroptosis and pyroptosis and identification of PTGS2 as the hub gene in human coronary artery atherosclerosis. *Free Radic Biol Med.* (2021) 171:55–68. 10.1016/j.freeradbiomed.2021.05.009 33974977

[32] Sakai O Yasuzawa T Sumikawa Y Ueta T Imai H Sawabe A Role of GPx4 in human vascular endothelial cells, and the compensatory activity of brown rice on GPx4 ablation condition. *Pathophysiology.* (2017) 24:9–15. 10.1016/j.pathophys.2016.11.002 27964880

[33] Sampilvanjil A Karasawa T Yamada N Komada T Higashi T Baatarjav C Cigarette smoke extract induces ferroptosis in vascular smooth muscle cells. *Am J Physiol Heart Circ Physiol.* (2020) 318:H508–18. 10.1152/ajpheart.00559.2019 31975626

[34] Li Z Kong W. Cellular signaling in abdominal aortic aneurysm. *Cell Signal.* (2020) 70:109575. 10.1016/j.cellsig.2020.109575 32088371

[35] Emeto TI Moxon JV Au M Golledge J. Oxidative stress and abdominal aortic aneurysm: Potential treatment targets. *Clin Sci.* (2016) 130:301–15. 10.1042/CS20150547 26814202

[36] Golledge AL Walker P Norman PE Golledge J. A systematic review of studies examining inflammation associated cytokines in human abdominal aortic aneurysm samples. *Dis Markers.* (2009) 26:181–8. 10.1155/2009/352319 19729799 PMC3833704

[37] Li J Krishna SM Golledge J. The Potential role of kallistatin in the development of abdominal aortic aneurysm. *Int J Mol Sci.* (2016) 17:1312. 10.3390/ijms17081312 27529213 PMC5000709

[38] Proell M Riedl SJ Fritz JH Rojas AM Schwarzenbacher R. The Nod-Like Receptor (NLR) family: A tale of similarities and differences. *PLoS One.* (2008) 3:e2119. 10.1371/journal.pone.0002119 18446235 PMC2323615

[39] Maitiseyiti A Ci H Fang Q Guan S Shawuti A Wang H Identification of novel long noncoding RNAs and their role in abdominal aortic aneurysm. *Biomed Res Int.* (2020) 2020:3502518. 10.1155/2020/3502518 33415145 PMC7769652

[40] Zhu T Shi L Yu C Dong Y Qiu F Shen L Ferroptosis promotes photodynamic therapy: Supramolecular photosensitizer-inducer nanodrug for enhanced cancer treatment. *Theranostics.* (2019) 9:3293–307. 10.7150/thno.32867 31244955 PMC6567978

[41] Tang R Xu J Zhang B Liu J Liang C Hua J Ferroptosis, necroptosis, and pyroptosis in anticancer immunity. *J Hematol Oncol.* (2020) 13:110. 10.1186/s13045-020-00946-7 32778143 PMC7418434

[42] Yee PP Li W. Tumor necrosis: A synergistic consequence of metabolic stress and inflammation. *Bioessays.* (2021) 43:e2100029. 10.1002/bies.202100029 33998010 PMC8217290

[43] Yuan Z Lu Y Wei J Wu J Yang J Cai Z. Abdominal aortic aneurysm: Roles of inflammatory cells. *Front Immunol.* (2020) 11:609161. 10.3389/fimmu.2020.609161 33613530 PMC7886696

[44] Nie H Qiu J Wen S Zhou W. Combining bioinformatics techniques to study the key immune-related genes in abdominal aortic aneurysm. *Front Genet.* (2020) 11:579215. 10.3389/fgene.2020.579215 33362847 PMC7758434

[45] Sagan A Mikolajczyk TP Mrowiecki W Macritchie N Daly K Meldrum A T cells are dominant population in human abdominal aortic aneurysms and their infiltration in the perivascular tissue correlates with disease severity. *Front Immunol.* (2019) 10:1979. 10.3389/fimmu.2019.01979 31552015 PMC6736986

[46] Suh MK Batra R Carson JS Xiong W Dale MA Meisinger T Ex vivo expansion of regulatory T cells from abdominal aortic aneurysm patients inhibits aneurysm in humanized murine model. *J Vasc Surg.* (2020) 72:1087–1096e1081. 10.1016/j.jvs.2019.08.285 31980239 PMC10690961

[47] Meng X Yang J Zhang K An G Kong J Jiang F Regulatory T cells prevent angiotensin II-induced abdominal aortic aneurysm in apolipoprotein E knockout mice. *Hypertension.* (2014) 64:875–82. 10.1161/HYPERTENSIONAHA.114.03950 25024283

[48] Zhou Y Wu W Lindholt JS Sukhova GK Libby P Yu X Regulatory T cells in human and angiotensin II-induced mouse abdominal aortic aneurysms. *Cardiovasc Res.* (2015) 107:98–107. 10.1093/cvr/cvv119 25824145 PMC4560044

[49] Liu B Kong J An G Zhang K Qin W Meng X. Regulatory T cells protected against abdominal aortic aneurysm by suppression of the COX-2 expression. *J Cell Mol Med.* (2019) 23:6766–74. 10.1111/jcmm.14554 31328426 PMC6787467

[50] Meng L Lu Y Wang X Sui W Ge X Zhong M Statin therapy protects against abdominal aortic aneurysms by inducing the accumulation of regulatory T cells in ApoE(–/–) mice. *J Mol Med.* (2022) 100:1057–70. 10.1007/s00109-022-02213-3 35704059

[51] Yang WS Sriramaratnam R Welsch ME Shimada K Skouta R Viswanathan VS Regulation of ferroptotic cancer cell death by GPX4. *Cell.* (2014) 156:317–31. 10.1016/j.cell.2013.12.010 24439385 PMC4076414

[52] Sutherland M Shankaranarayanan P Schewe T Nigam S. Evidence for the presence of phospholipid hydroperoxide glutathione peroxidase in human platelets: Implications for its involvement in the regulatory network of the 12-lipoxygenase pathway of arachidonic acid metabolism. *Biochem J.* (2001) 353:91–100. 10.1042/bj3530091 11115402 PMC1221546

[53] Strauss E Tomczak J Staniszewski R Oszkinis G. Associations and interactions between variants in selenoprotein genes, selenoprotein levels and the development of abdominal aortic aneurysm, peripheral arterial disease, and heart failure. *PLoS One.* (2018) 13:e0203350. 10.1371/journal.pone.0203350 30188935 PMC6126836

[54] Xie M Huang P Wu T Chen L Guo R. Inhibition of miR-214-3p protects endothelial cells from ox-LDL-induced damage by targeting GPX4. *Biomed Res Int.* (2021) 2021:9919729. 10.1155/2021/9919729 34327240 PMC8277498

[55] Yang Q Xu J Ma Q Liu Z Sudhahar V Cao Y PRKAA1/AMPKalpha1-driven glycolysis in endothelial cells exposed to disturbed flow protects against atherosclerosis. *Nat Commun.* (2018) 9:4667. 10.1038/s41467-018-07132-x 30405100 PMC6220207

[56] Song X Liu J Kuang F Chen X Zeh HJ III Kang R PDK4 dictates metabolic resistance to ferroptosis by suppressing pyruvate oxidation and fatty acid synthesis. *Cell Rep.* (2021) 34:108767. 10.1016/j.celrep.2021.108767 33626342

[57] Keller ET. Metastasis suppressor genes: A role for raf kinase inhibitor protein (RKIP). *Anticancer Drugs.* (2004) 15:663–9. 10.1097/01.cad.0000136877.89057.b9 15269597

[58] Qin Q Liu H Shou J Jiang Y Yu H Wang X. The inhibitor effect of RKIP on inflammasome activation and inflammasome-dependent diseases. *Cell Mol Immunol.* (2021) 18:992–1004. 10.1038/s41423-020-00525-3 32901127 PMC8115060

[59] Hu X Wu P Liu B Lang Y Li T. RNA-binding protein CELF1 promotes cardiac hypertrophy via interaction with PEBP1 in cardiomyocytes. *Cell Tissue Res.* (2021) 387:111–21. 10.1007/s00441-021-03541-5 34669021

[60] Wang L Li H Zhang J Lu W Zhao J Su L Phosphatidylethanolamine binding protein 1 in vacular endothelial cell autophagy and atherosclerosis. *J Physiol.* (2013) 591:5005–15. 10.1113/jphysiol.2013.262667 23959677 PMC3810805

