# Supplementary figures and images for: Key ferroptosis-related genes in abdominal aortic aneurysm formation and rupture as determined by combining bioinformatics techniques
Source: Front Cardiovasc Med. 2022 Aug 9;9:875434. doi: 10.3389/fcvm.2022.875434 (PMC9395677; doi:10.3389/fcvm.2022.875434)

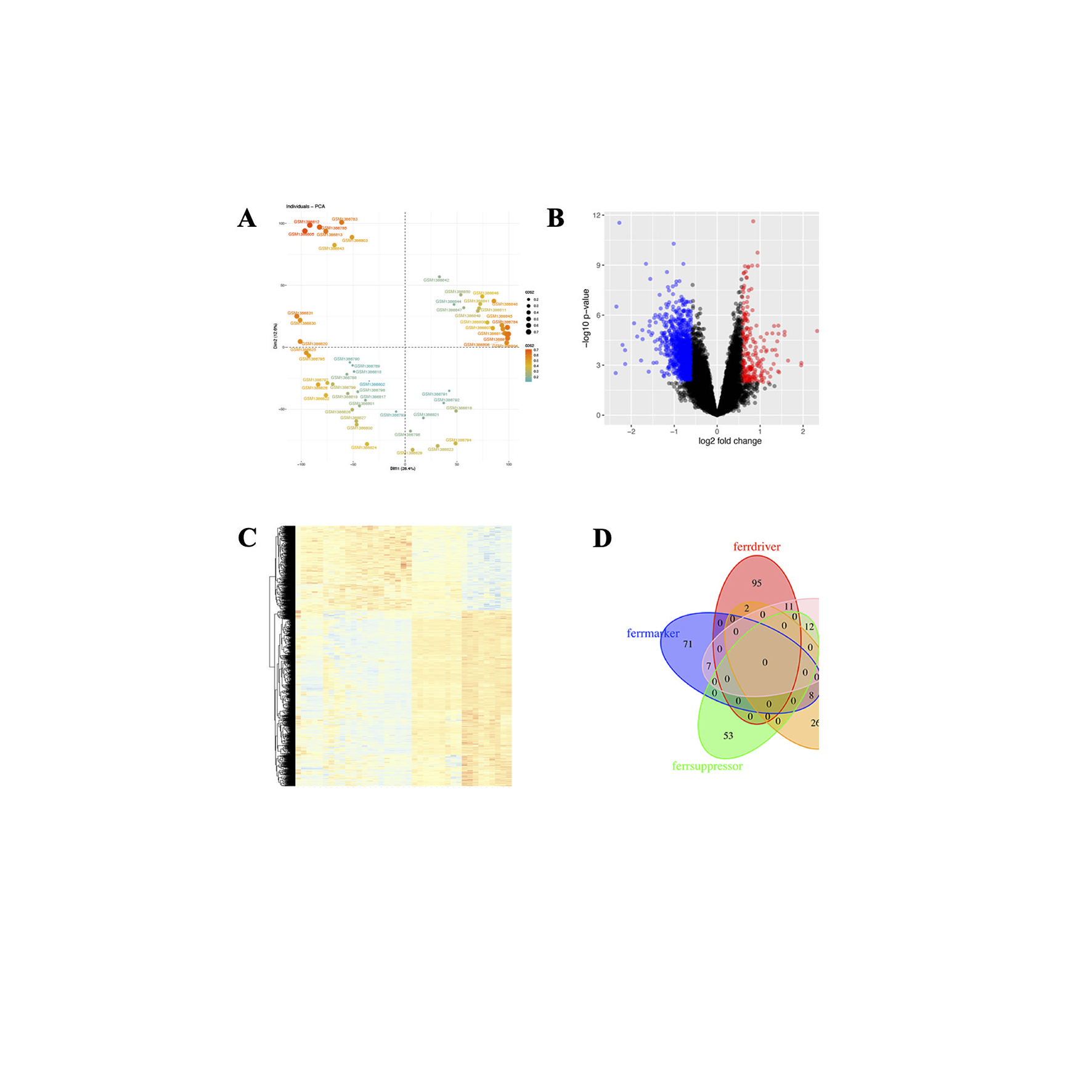

Supplement: Supplementary file 4 [file Image_1.TIFF]

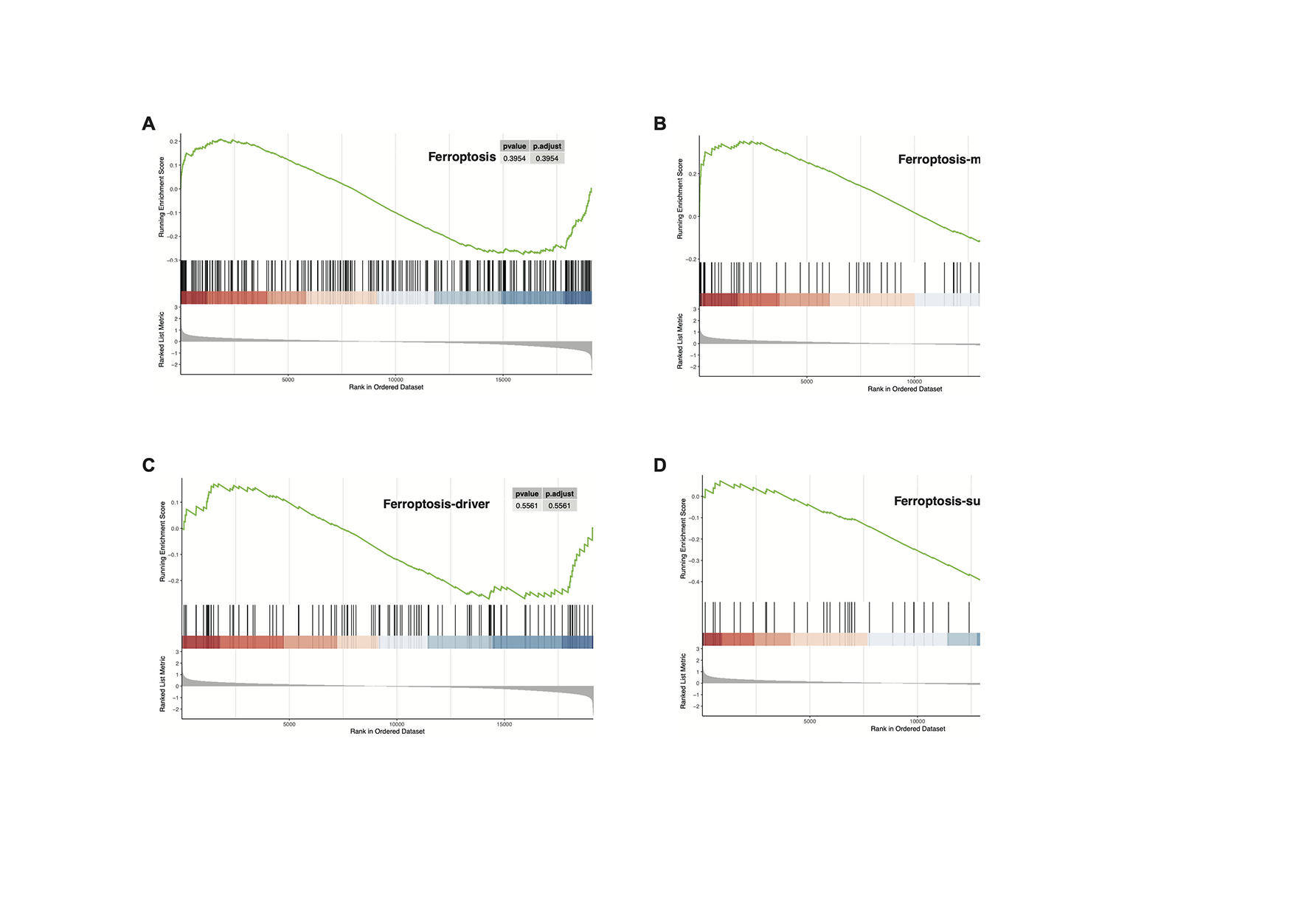

Supplement: Supplementary file 5 [file Image_2.TIFF]

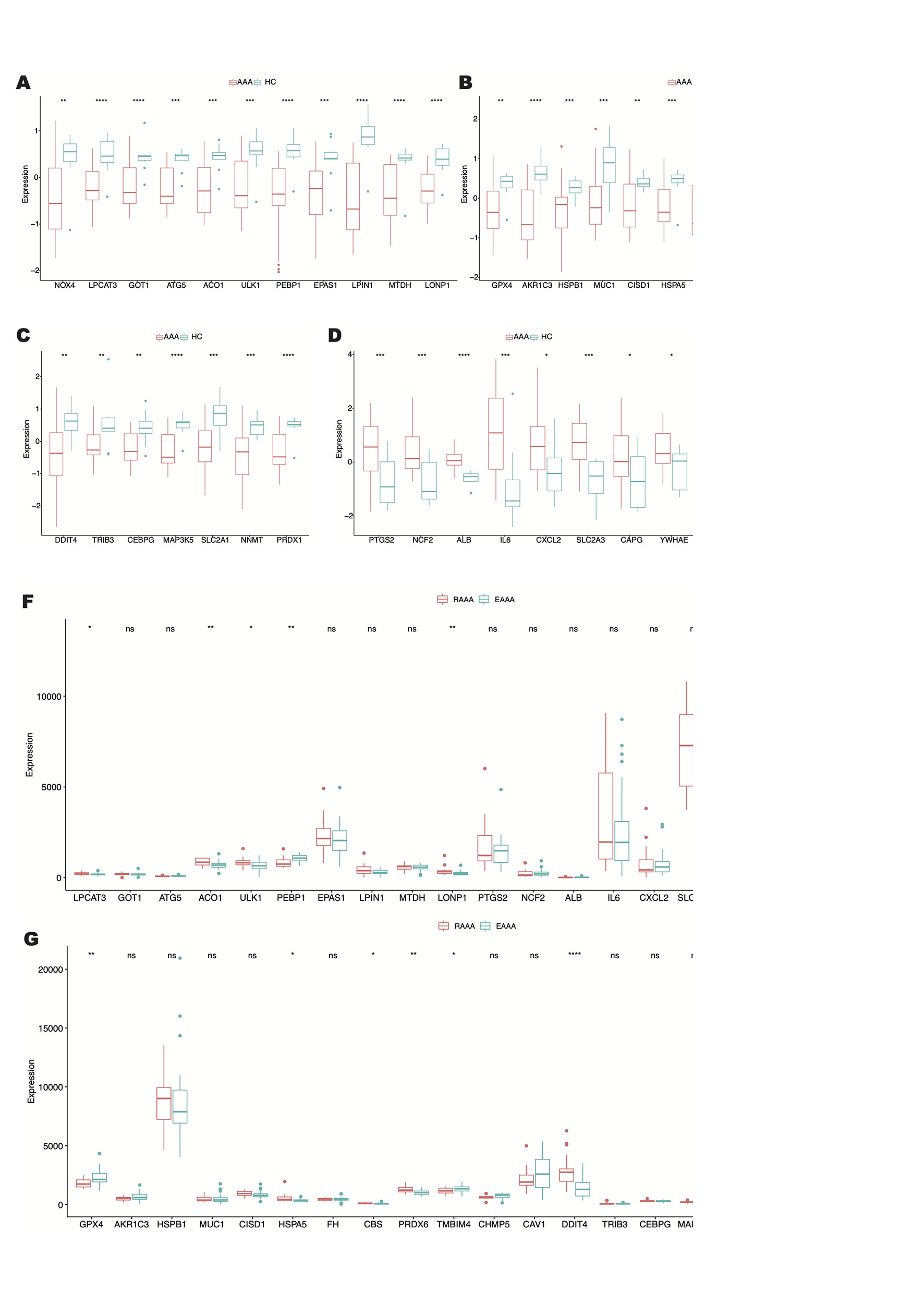

Supplement: Supplementary file 6 [file Image_3.TIFF]
